# Polyphenols and Flavonoids in microRNA-Regulated Pathways of Doxorubicin-Induced Cardiotoxicity: Mechanistic Evidence, Biomarker Potential and Translational Gaps

**DOI:** 10.3390/ijms27209224

**Published:** 2026-10-17

**Authors:** Dimitra Ioanna Lampropoulou, Antigoni Kouskouti, Aristea Gioxari, Evangelia Pliakou, Konstantina Argyri, Vasileios Dedes, George Panoutsopoulos, Maria Gazouli

**Affiliations:** 1 Department of Nutritional Science and Dietetics, Faculty of Health Sciences, University of Peloponnese, 24100 Kalamata, Greece; a.kouskouti@uop.gr (A.K.); a.gioxari@uop.gr (A.G.); kargyri@uop.gr (K.A.); v.dedes@uop.gr (V.D.); gpanouts@uop.gr (G.P.); 2 Department of Medical Oncology, Athens Naval and Veterans Hospital, 11521 Athens, Greece; evangeliaplk@hotmail.com; 3 Laboratory of Biology, Department of Basic Medical Sciences, Medical School, National and Kapodistrian University of Athens, Michalakopoulou 176, 11527 Athens, Greece; mgazouli@med.uoa.gr

**Keywords:** anthracycline cardiotoxicity, biomarker, cardio-oncology, flavonoids, microRNAs, polyphenols

## Abstract

Although doxorubicin remains a cornerstone of anticancer therapy, its clinical utility is limited by dose-dependent cardiotoxicity. Current tools for the early detection of doxorubicin-induced cardiotoxicity (DIC) remain suboptimal, prompting interest in microRNAs (miRNAs) as mechanistic regulators and circulating biomarkers. Natural cardioprotective compounds, particularly polyphenols and flavonoids, have also attracted attention because of their ability to modulate oxidative stress, mitochondrial dysfunction, inflammation, autophagy and regulated cell death. However, the mechanistic and translational strength of this evidence varies considerably. This critical narrative review is based on targeted PubMed literature searches up to 14 August 2026, supplemented by selective reference-list screening of relevant original articles and reviews. The literature search was not intended to be exhaustive, and the studies discussed were selected to illustrate different levels of mechanistic evidence. Studies investigating miRNA-mediated mechanisms in DIC were distinguished from circulating biomarker studies, while phytochemical evidence was classified as direct miRNA-linked, miRNA-associated without causal validation, or pathway-convergent. Among the selected examples, taxifolin provides direct miRNA-linked evidence involving the miR-200a/nuclear factor erythroid 2-related factor 2 (NRF2) pathway, with functional miR-200a inhibition demonstrated in H9c2 cells. Blueberry extract provided miRNA-associated evidence; miR-140-5p expression was assessed together with *Sirt2* and *Nrf2* expression, but a causal role for miR-140-5p was not functionally validated. For the other selected compounds, the available evidence was predominantly pathway-convergent. Although miRNAs and phytochemicals offer complementary perspectives on DIC mechanisms and cardioprotection, their clinical application remains premature. Future studies require functional miRNA validation, standardized biomarker assessment, pharmacokinetic evaluation and confirmation that cardioprotection does not compromise antitumor efficacy.

## 1. Introduction

Doxorubicin represents one of the most effective and widely used anthracyclines in solid tumors and hematologic malignancies for more than 50 years. It remains an important component of treatment regimens for several malignancies, including breast cancer (BC), lymphomas, leukemias and sarcomas [1,2,3]. Its antitumor activity is mainly mediated by stabilizing the topoisomerase II–DNA cleavage complex, leading to DNA damage and ultimately cell death [3]. However, dose-dependent cardiotoxicity remains one of its most challenging and clinically relevant toxicities, compromising long-term survivorship even when cancer control is achieved [1,2]. Indeed, the risk of cardiac injury increases with cumulative doxorubicin exposure, although clinically relevant cardiotoxicity may also occur at lower cumulative doses in susceptible patients [1,2,3]. The recommended maximum lifetime cumulative dose of doxorubicin should not exceed 400–550 mg/m^2^ [4].

Anthracycline-related cardiac dysfunction encompasses a broad clinical spectrum. Contemporary cardio-oncology definitions distinguish asymptomatic from symptomatic cancer therapy-related cardiac dysfunction (CTRCD). Mild asymptomatic CTRCD may occur despite preserved left ventricular ejection fraction (LVEF) and can be identified by a >15% relative decline in global longitudinal strain (GLS) and/or a new increase in cardiac biomarkers. More severe asymptomatic forms involve a reduction in LVEF, whereas symptomatic CTRCD presents with signs or symptoms of heart failure [1,2]. Therefore, biomarker elevation and subclinical cardiac dysfunction should be distinguished from clinically manifest cardiotoxicity.

Improved cancer survivorship has made anthracycline toxicity an increasingly important clinical concern, with a growing burden of associated morbidity and mortality. Current cardio-oncology guidelines focus on risk stratification, baseline cardiac evaluation, surveillance and timely intervention [1,2]. Biomarkers, such as cardiac troponin and B-type natriuretic peptide (BNP), as well as echocardiographic measures, such as GLS, have improved surveillance strategies but remain suboptimal to capture the full spectrum of early cardiotoxicity. For example, elevated troponin may indicate early myocardial injury, whereas imaging abnormalities may emerge later in the course of cardiac dysfunction [1,2].

In light of the above, scientific interest has focused on circulating miRNAs as potential biomarkers for the early detection of doxorubicin-induced cardiac dysfunction. MicroRNAs (miRNAs) are key post-transcriptional regulators involved in multiple cellular responses underlying doxorubicin-induced cardiotoxicity (DIC) [5,6,7]. Notably, their relative stability in plasma and the feasibility for repeated measurements make them attractive candidate biomarkers [5,6,7,8,9].

In parallel, increasing interest has developed around natural cardioprotective compounds, especially polyphenols and flavonoids. Polyphenols are plant-derived bioactive compounds that include several subclasses, among which flavonoids are extensively studied [10]. These phytochemicals have been linked to several mechanisms relevant to DIC, including oxidative stress, preservation of mitochondrial integrity, inflammation, autophagy and regulated cell death [11]. However, their translational relevance remains heterogeneous in the current literature. Although many studies report a cardioprotective signal, they often do not establish whether this protection is directly mediated by a specific miRNA.

On this basis, the present review evaluates selected evidence on mechanistic miRNA pathways, circulating biomarkers and phytochemical cardioprotection. The discussion focuses on the strength of the mechanistic evidence, the distinction between direct and pathway-based findings, and the main gaps that currently limit clinical translation.

## 2. Scope and Approach of This Critical Narrative Review

This critical narrative review examines three interconnected areas: (i) molecular mechanisms of doxorubicin-induced cardiotoxicity (DIC) relevant to miRNA regulation, (ii) experimental and clinical evidence regarding miRNAs in DIC, and (iii) selected examples of polyphenol- and flavonoid-mediated cardioprotection that illustrate different degrees of mechanistic proximity to miRNA-regulated pathways. Relevant literature was identified through targeted PubMed searches, up to 14 August 2026, supplemented by selective reference-list screening of relevant original articles and reviews. Search terms included combinations of “doxorubicin” or “anthracycline” with “cardiotoxicity”, “microRNA” or “miRNA”, “biomarker”, “polyphenol”, “flavonoid”, and individual compound names. Studies were selected as representative examples of the existing evidence, covering mechanistic miRNA findings, circulating biomarkers, and phytochemical cardioprotection. For the phytochemical section, studies were chosen to illustrate different types of relationships between cardioprotective effects and miRNA-regulated mechanisms in DIC. These were classified as: (i) direct miRNA-linked evidence, when miRNA involvement was functionally investigated in the context of phytochemical cardioprotection; (ii) miRNA-associated evidence, when changes in miRNA expression were reported without functional validation of causality; and (iii) pathway-convergent evidence, when the experimentally observed cardioprotective effects overlapped with pathways known to be regulated by DIC-associated miRNAs, without, however, direct assessment of miRNA involvement. These categories are used descriptively and should not be interpreted as a formal hierarchy of methodological quality or risk of bias. Given the critical narrative design of this review, no formal risk-of-bias tool was applied; instead, methodological limitations relevant to interpretation of the evidence are discussed qualitatively throughout the review.

## 3. Doxorubicin-Induced Cardiotoxicity: Mechanistic Background

The mechanisms underlying DIC are not fully understood and are likely to involve multiple and mutually interacting pathways [3,4]. The major proposed mechanisms have been broadly grouped into nuclear- and mitochondrial-mediated pathways [4].

Nuclear-mediated DIC is associated with the interaction of doxorubicin with topoisomerase IIβ in cardiomyocytes [4,12]. This disruption promotes DNA double-strand breaks and dysfunctional transcriptional responses that compromise mitochondrial biogenesis and cellular homeostasis [12]. These transcriptional alterations contribute to mitochondrial dysfunction, while topoisomerase IIβ-mediated DNA damage promotes downstream pro-apoptotic signaling [4,12]. Supporting the importance of nuclear injury, Jean et al. examined a mitochondria-targeted doxorubicin derivative that lacked direct nuclear effects. According to the authors, this derivative preserved a compensatory mitochondrial biogenesis response and reduced cell death in H9c2 cardiomyocytes [13]. Moreover, topoisomerase IIβ deletion in cardiomyocytes has been associated with protection from doxorubicin-induced DNA damage, transcriptomic dysregulation, mitochondrial defects and progressive heart failure [12].

Another key mechanistic axis in DIC is mitochondrial dysfunction. Doxorubicin disrupts mitochondrial homeostasis at multiple levels, including electron transport dysfunction, mitochondrial membrane depolarization, altered mitochondrial biogenesis and overproduction of reactive oxygen species (ROS) [3,14,15]. More specifically, adriamycin derivatives inhibit mitochondrial complex I-III through a cardiolipin-dependent mechanism [16]. Doxorubicin also increases mitochondrial membrane permeability, promoting cytochrome c release and caspase-3 activation, thereby contributing to mitochondria-mediated apoptosis [15]. In addition, dysregulated iron homeostasis can amplify oxidative stress and promote lipid peroxidation, contributing to ferroptosis in doxorubicin-induced cardiac injury [11,15]. At the same time, anthracyclines can increase the mitochondrial membrane permeability leading to (i) impaired mitochondrial function, (ii) reduced cellular respiration and (iii) promotion of regulated cell-death pathways, such as necroptosis [11,17]. Increased mitochondrial membrane permeability can also promote the release of apoptosis-inducing factor (AIF), which mediates caspase-independent cell death [4].

The different mechanisms of injury in DIC do not act independently; on the contrary, they appear to interact within a cardiac injury network, and this is why miRNAs become biologically important. In DIC, miRNAs often serve as regulatory mediators, connecting upstream stress signals with downstream phenotypes, such as oxidative damage, mitochondrial dysfunction, impaired survival signaling, fibrosis and endothelial dysfunction [8,18,19,20,21]. A single miRNA may affect several components of the doxorubicin-induced injury cascade, while multiple miRNAs may act on the same pathogenic axis. The role of distinct miRNA signatures in DIC is further discussed in the following section of the review.

This mechanistic complexity also provides a strong rationale for exploring phytochemical cardioprotection in DIC. The potential value of polyphenols and flavonoids lies in their ability to modulate the broader stress-response network of the injured myocardium. A consistent finding across the preclinical studies considered in this review is that these compounds, despite their chemical heterogeneity, tend to result in relatively common protective phenotypes, i.e., reduction in oxidative injury and cellular apoptosis and preservation of mitochondrial homeostasis [11]. However, this mechanistic range also creates an important translational challenge: A compound may be linked with a protective phenotype, but its precise mechanism of action remains unclear unless a specific miRNA-regulated axis is directly investigated. This distinction is central to the present review, since functional evidence linking phytochemical cardioprotection to a specific miRNA remains limited.

## 4. miRNAs in DIC

**Figure 1:**
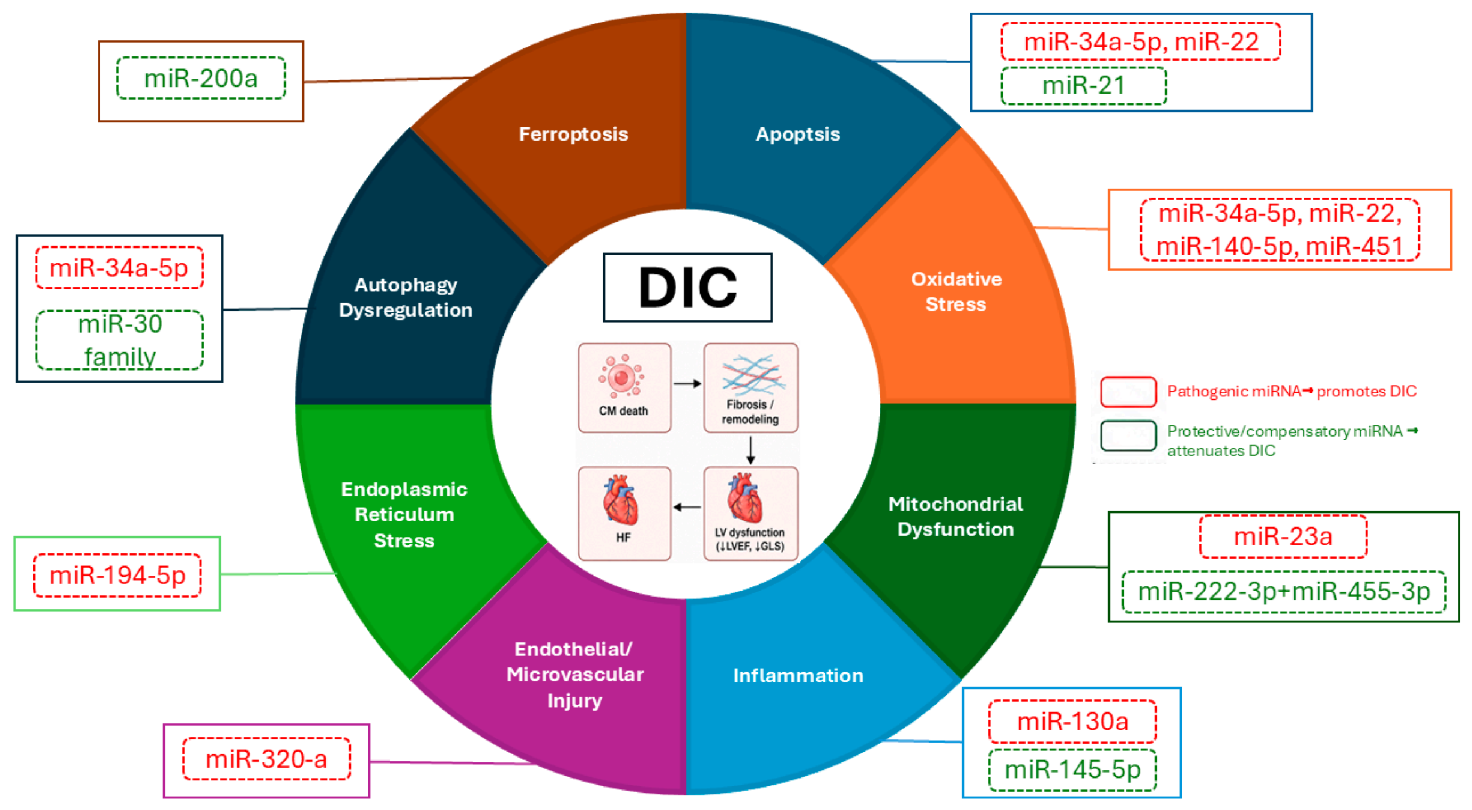
Major pathogenic processes and representative miRNAs implicated in doxorubicin-induced cardiotoxicity. Abbreviations: DIC, doxorubicin-induced cardiotoxicity; CM, cardiomyocyte; HF, heart failure; LV, left ventricle; LVEF, left ventricular ejection fraction; GLS, global longitudinal strain, miRNA, microRNA. Figure 1 Note: Red labels indicate miRNAs with predominantly pathogenic effects, whereas green labels indicate protective or compensatory miRNAs in the DIC models discussed. Supporting evidence includes miR-34a-5p [25,26,27], miR-22 [28], miR-140-5p [29], miR-21 [30,31,32], miR-30 family [33,34], miR-451 [35], miR-23a [36], miR-222-3p/miR-455-3p [37], miR-145-5p [38], miR-320a [39], miR-194-5p [40], miR-130a [41], and miR-200a [42]. This classification reflects the main, experimentally observed effect in the cited studies and may vary according to the experimental context.

miRNAs are short non-coding RNAs that downregulate gene expression post-transcriptionally. Their implication in DIC involves several processes including the regulation of apoptosis, mitochondrial homeostasis, redox signaling, fibrosis, angiogenesis, autophagy, ferroptosis and inflammatory pathways [5,6,7,8,9] (Figure 1). However, the functional effects of individual miRNAs may vary according to cell type, experimental model, disease stage and other biological or experimental conditions. Accordingly, their classification as predominantly pathogenic or protective in Figure 1 reflects the effects observed in the DIC models discussed in this review rather than fixed biological properties.

For clarity, this review distinguishes between two partially overlapping bodies of literature. The first group includes preclinical studies of myocardial/cellular miRNAs in cell-based and animal models, focusing on their mechanistic roles and causal relevance in DIC. The second concerns circulating miRNA studies in humans that examine whether specific miRNAs may serve as minimally invasive biomarkers of anthracycline-related injury. A miRNA that is mechanistically important in the myocardium may not necessarily be a useful circulating biomarker, whereas a circulating miRNA associated with DIC may not itself be a key driver of myocardial injury [21,22,23,24]. Accordingly, Section 4.1 focuses on myocardial and cellular miRNAs with experimentally supported mechanistic roles in DIC, whereas Section 4.2 addresses circulating miRNAs evaluated as potential clinical biomarkers. Overlap between the two evidence groups is highlighted only when supported by both experimental and clinical data.

### 4.1. Mechanistic Myocardial and Cellular miRNAs in DIC

One of the most discussed axes in the current literature is miR-34a/sirtuin 1 (SIRT1). Doxorubicin upregulates miR-34a-5p, which targets SIRT1, and thereby enhances 66 kDa Shc adaptor protein (p66Shc)-mediated pro-apoptotic and oxidative stress signaling. This pathway contributes to oxidative stress and cardiomyocyte apoptosis [25]. Accordingly, miR-34a inhibition reduces cardiomyocyte death and attenuates left ventricular dysfunction in experimental DIC [26]. Overall, miR-34a-5p has been suggested as an important regulator of senescence, redox imbalance and apoptosis in the injured myocardium [25,26].

miR-22 is another pathogenic miRNA with strong relevance to oxidative stress and apoptosis. miR-22 inhibition has been reported to reduce doxorubicin-induced injury via SIRT1 upregulation and reduction in oxidative stress [28]. Taken together, miR-34a and miR-22 participate in a SIRT1-associated regulatory axis that links oxidative stress and survival signaling in DIC [14,26,28].

A similarly important redox pathway involves miR-140-5p; doxorubicin-induced upregulation of miR-140-5p promotes DIC through targeting nuclear factor erythroid 2-related factor 2 (*Nrf2*) and sirtuin 2 (*Sirt2*). This results in increased oxidative injury and impaired endogenous antioxidant defense, particularly through the disruption of NRF2-dependent cytoprotective responses. On the contrary, silencing miR-140-5p restores NRF2 signaling, reduces oxidative stress and attenuates myocardial injury [29].

On the other hand, some DIC-associated miRNAs have been linked with endogenous cardioprotective responses. For example, miR-21 has been shown to protect cardiomyocytes from doxorubicin-induced apoptosis via B-cell translocation gene 2 (BTG2)-related mechanisms [30,31]. Although miR-21 can be context-dependent in cardiovascular biology, its upregulation through nucleolin has been suggested to protect cardiomyocytes from doxorubicin-induced apoptosis, whereas its inhibition favors cell death [32].

The miR-30 family appears to play a broader role in survival signaling. Myocardial miR-30 downregulation following doxorubicin administration has been linked to altered β-adrenergic signaling and enhanced apoptosis [33]. Moreover, miR-30e has been reported to contribute to angiotensin-converting enzyme 2 (ACE2)-mediated cardioprotection via the suppression of autophagy [34].

AMP-activated protein kinase (AMPK)-dependent stress responses in DIC are also regulated by miRNAs. Silencing of miR-451 reduced oxidative stress, apoptosis and cardiac dysfunction by relieving calcium binding protein 39 (*Cab39*) suppression and by activating AMPK; pharmacological AMPK inhibition abolished the protective effect on cell viability in vitro [35]. In addition, inhibition of miR-34a-5p restored the autophagic balance and reduced myocardial pyroptosis. These effects involved the sirtuin 3 (SIRT3)/AMPK axis [27].

Regarding mitochondrial function, miR-23a promotes doxorubicin-induced mitochondrial injury by directly suppressing the peroxisome proliferator-activated receptor gamma coactivator 1-alpha (PGC-1α). Inhibition of miR-23a preserved the mitochondrial membrane potential and reduced ROS generation and apoptosis. Silencing of PGC-1α reversed these protective effects [36].

In addition to individual miRNAs, Higashikuni et al. identified miR-222-3p and miR-455-3p as a synergistic combination that improved cardiomyocyte survival and reduced DIC phenotypes in zebrafish and mice [37].

Other mechanistically important miRNAs extend the spectrum of DIC beyond the oxidative stress and apoptotic pathways described above. Restoration of miR-145-5p attenuated cardiomyocyte pyroptosis through the SRY-box transcription factor 9 (SOX9)/NOD-, LRR- and pyrin domain-containing protein 3 (NLRP3) inflammasome axis [38]. Furthermore, miR-320a has been implicated in impaired vascular homeostasis through vascular endothelial growth factor (VEGF) signaling [39]. miR-194-5p has been associated with a pathogenic role in DIC through suppression of PAK2 and modulation of the PAK2/XBP1s axis. Its inhibition reduced doxorubicin-induced endoplasmic reticulum stress and cardiomyocyte apoptosis [40]. miR-130a has also been associated with inflammatory signaling and apoptosis [41]. Collectively, these findings broaden the mechanistic spectrum of DIC to include pyroptosis, endothelial dysfunction, endoplasmic reticulum stress, inflammation and metabolic dysregulation. It should be noted that these experimental studies support mechanistic roles for specific miRNAs in DIC, but such evidence does not by itself establish their utility as circulating clinical biomarkers.

### 4.2. Circulating miRNAs as Candidate Biomarkers in DIC

Although circulating miRNAs are being investigated as possible early biomarkers of DIC, the current literature remains heterogeneous [18,19,20]. Several circulating candidates have been investigated in human studies, including miR-1, miR-133a, let-7 family members, miR-20a, miR-126, miR-210 and miR-199a. However, results remain inconsistent and currently no single miRNA is sufficiently validated for clinical implementation [18,19,20,21]. Within this broader anthracycline literature, the clinical evidence, specifically derived from doxorubicin-treated patients, remains even more limited and heterogeneous. In a prospective BC cohort treated with doxorubicin, circulating miR-1 was associated with changes in LVEF and showed an area under the curve (AUC) of 0.851 for cardiotoxicity [43]. In a small case–control study, serum miR-133a was markedly increased at the end of treatment in women who developed DIC, although no diagnostic threshold or independent validation was reported [44]. More recently, plasma miR-199a increased during dose-dense doxorubicin/cyclophosphamide treatment, but changes in miR-199a were not significantly associated with contemporaneous changes in LVEF [45]. In addition, Gioffré et al. reported that a baseline circulating miRNA signature was associated with subsequent troponin elevation in doxorubicin-treated BC patients; however, this study primarily evaluated subclinical myocardial injury rather than imaging-defined cardiotoxicity [24]. It should be noted that these studies assessed different biomarker outcomes. Gioffré et al. evaluated the prediction of troponin elevation [24]; Rigaud et al. assessed the ability of miR-1 to identify patients with cardiotoxicity [43]; Alves et al. reported differences in miRNA expression at the end of treatment between patients with and without cardiotoxicity [44]; and Kamian et al. evaluated treatment-related changes in miR-199a expression [45]. None of them included validation in an independent external cohort. Therefore, these findings do not establish clinical utility and should be interpreted cautiously. Key methodological and clinical characteristics of the primary doxorubicin-based circulating-miRNA studies discussed above are summarized in Supplementary Table S1.

The current heterogenous evidence may reflect differences in anthracycline regimens and cumulative exposure, definitions of cardiotoxicity, biological matrix and sampling timepoints, as well as inconsistent normalization strategies across miRNA assays [18,20,21]. Therefore, the literature on circulating biomarkers should be distinguished from the mechanistic evidence discussed in Section 4.1, where miRNAs are considered primarily as regulators of DIC pathways rather than as clinically validated circulating biomarkers. Large-scale, standardized prospective studies are needed before any of the proposed miRNAs can be incorporated into routine cardio-oncology practice. At present, the most reasonable interpretation is that circulating miRNAs may be valuable candidates at a research level and may ultimately perform best as panels integrated with troponin, BNP and imaging [18,20,21].

## 5. Polyphenol- and Flavonoid-Mediated Cardioprotection in DIC: Relationships with miRNA-Regulated Pathways

The relationship between phytochemical cardioprotection and miRNA-regulated mechanisms in DIC can be viewed at different levels of mechanistic support. Among the examples considered in this review, some studies directly investigated miRNA involvement during phytochemical treatment. Others reported changes in miRNA expression without functional validation of causality. A third group showed mechanistic convergence between cardioprotective effects and pathways also regulated by DIC-associated miRNAs, without direct assessment of miRNA involvement. The phytochemicals discussed below represent selected examples and should not be interpreted as an exhaustive inventory of polyphenols or flavonoids investigated in DIC.

### 5.1. Taxifolin (Dihydroquercetin)

Taxifolin is an example with direct miRNA-linked evidence involving the miR-200a/NRF2 pathway. Taxifolin is a natural flavonoid found in citrus fruits, coniferous plants, onions and *Ginkgo biloba* with anti-inflammatory, anti-apoptotic and antioxidant properties [42,46]. Lin et al. investigated taxifolin in both a mouse model of DIC and doxorubicin-exposed H9c2 cells. Taxifolin increased miR-200a expression and activated the Kelch-like ECH-associated protein 1 (KEAP1)/NRF2 pathway. Importantly, inhibition of miR-200a in H9c2 cells reduced NRF2 signaling and partially reversed the protective effects of taxifolin on cytotoxicity, oxidative stress and ferroptosis. *Nrf2* silencing also attenuated taxifolin-mediated protection. These functional experiments in H9c2 cells support the involvement of the miR-200a/KEAP1/NRF2 axis in taxifolin-mediated protection [42]. However, findings from the mouse model demonstrate cardioprotection without directly showing that miR-200a mediates this effect in vivo.

### 5.2. Blueberry Extract

Blueberry extract is a polyphenol-rich preparation derived from *Vaccinium* berries. It contains anthocyanins, flavonols and phenolic acids with antioxidant and anti-inflammatory properties [47]. In a rat DIC model, blueberry extract attenuated biochemical and histopathological injury and restored cardiac *Sirt2* and *Nrf2* expression. Cardiac miR-140-5p expression was also assessed, although the statistical significance of its treatment-related changes was inconsistently reported [48]. However, miR-140-5p was not manipulated using gain- or loss-of-function or rescue experiments, and thus its causal involvement in blueberry-mediated cardioprotection remains unproven.

### 5.3. Quercetin

Quercetin, a widely distributed dietary flavonol found in onions, fruits, leafy vegetables and tea, has been investigated in preclinical DIC models. An earlier study showed reduction in doxorubicin cardiotoxicity through B-cell-specific moloney murine leukemia virus integration site 1 (Bmi-1) regulation [49]; subsequent work has linked quercetin with oxidative stress suppression and improved mitochondrial function via 14-3-3γ-associated signaling [50]. More recent in vivo rat studies further support the cardioprotective activity of quercetin. One study reported improvements in cardiac ultrasound parameters and serum markers [51], while another showed attenuation of oxidative and histological damage along with differential modulation of p53 immunoexpression in myocardial fibers and cardiac fibroblasts [52]. However, none of these studies demonstrated a direct miRNA-mediated mechanism.

### 5.4. Resveratrol

Resveratrol is a polyphenol found in red wine, grapes, berries and peanuts [53,54]. Several studies have supported its cardioprotective activity in DIC, without directly investigating whether this protection depends on direct manipulation of a defined miRNA. The observed effects may be linked to SIRT1-related signaling, preservation of mitochondrial function, attenuation of p53-dependent apoptosis and modulation of autophagy [53,54,55,56]. These pathways overlap with stress-response mechanisms that are also regulated by DIC-associated miRNAs.

### 5.5. Curcumin

Curcumin is a polyphenol found in turmeric with antioxidant, anti-inflammatory and cardioprotective effects [57]. In vitro and in vivo studies support the cardioprotective effect of curcumin in DIC. Reported findings include reduced oxidative stress, preserved mitochondrial function, increased 14-3-3γ expression, as well as anti-inflammatory and anti-apoptotic effects [57,58,59].

### 5.6. Epigallocatechin Gallate (EGCG)

EGCG is a green tea-derived polyphenol [60]. In the studies considered here, its cardioprotective effects in DIC were associated with reduced oxidative stress, preservation of mitochondrial function, and attenuation of ferroptosis through the upregulation of AMP-activated protein kinase alpha 2 (AMPKα2) and adaptive autophagy [61]. These studies did not directly assess whether a specific miRNA mediates the observed cardioprotection [60,61,62,63].

### 5.7. Isoquercitrin

Isoquercitrin is a flavonoid found abundantly in fruits, vegetables and medicinal herbs, with reported cardioprotective effects [64,65]. An integrated network pharmacology and RNA sequencing (RNA-seq) analysis identified molecular changes associated with reduced oxidative stress and inflammation [65].

### 5.8. Genistein

Genistein is a phytoestrogen found in plants, particularly soybeans [10]. In preclinical DIC studies, genistein improved antioxidant defense and reduced cardiac injury [66]. It was also associated with lower levels of maladaptive autophagy and apoptosis [67]. Notably, Wu et al. reported that the higher genistein dose (40 mg/kg/day) was less protective and showed potentially unfavorable cardiac effects [67]. This finding suggests that the cardioprotective effects of genistein may be dose dependent.

### 5.9. Additional Pathway-Convergent Examples

Recent studies have broadened the pathway-convergent category. Engeletin attenuated doxorubicin-induced cardiac dysfunction, fibrosis, apoptosis, oxidative stress, inflammation, autophagy dysregulation and mitochondrial injury through AMPK activation [68]. Functional involvement of AMPK was demonstrated experimentally, but no specific miRNA was assessed as part of this protective mechanism. Total flavonoids from *Dracocephalum moldavica* L. preserved mitochondrial homeostasis through the AMPK/PGC-1α signaling pathway [69]. The latter mechanism was experimentally supported, but miRNA involvement was not investigated [69].

Table 1 summarizes the selected phytochemical examples by showing which miRNA was directly assessed and whether functional miRNA validation was performed. Furthermore, it presents the experimentally supported cardioprotective mechanism and the corresponding critical interpretation.

Key experimental characteristics of the phytochemical studies discussed above are summarized in Supplementary Table S2. The table provides a qualitative overview of selected methodological and reporting features and does not present a structured study-level quality or risk-of-bias assessment.

The cardioprotective actions of some of the phytochemicals discussed above have also been investigated beyond DIC. For example, quercetin and resveratrol have shown protective effects in models of myocardial ischemia–reperfusion injury. These effects have been linked to attenuation of oxidative stress, preservation of mitochondrial function and modulation of pathways including SIRT1, NRF2 and AMPK. This partial mechanistic overlap suggests that some cardioprotective effects may extend across different forms of cardiac injury, although the initiating mechanisms and experimental contexts are distinct [70].

## 6. Translational Limitations of Phytochemical Cardioprotection in DIC

Overall, a recurring limitation of the studies discussed in this review is that the evidence for phenotypic cardioprotection by polyphenols and flavonoids accumulates more rapidly than the evidence of mechanistic validation. Indeed, most of the phytochemical studies considered in this review report reduced ROS, less apoptosis, improved histology and/or preserved cardiac function; however, very few examine whether a specific miRNA is implicated in the protective effect using gain- and loss-of-function approaches. Therefore, mechanistic conclusions often remain suggestive rather than definitive even when the cardioprotective phenotype is convincing.

A further translational limitation concerns pharmacokinetics and systemic exposure. For example, curcumin, resveratrol, quercetin and EGCG undergo extensive first-pass metabolism and show limited or highly variable systemic exposure, although their absorption and metabolic profiles differ substantially. Curcumin has particularly low systemic bioavailability due to limited absorption and extensive reduction, glucuronidation and sulfation [71,72]. Resveratrol is relatively well absorbed, but rapid first-pass conjugation results in very low circulating concentrations of unchanged resveratrol [73]. Quercetin disposition depends strongly on its chemical form and the food matrix, while circulating quercetin is present predominantly as conjugated metabolites rather than as the parent aglycone [74]. EGCG also shows limited and variable oral bioavailability, with systemic exposure influenced by formulation and food intake [75]. These differences may be particularly important since the cardioprotective effects observed with high micromolar concentrations of parent compounds in vitro may not reflect clinically attainable exposure after oral administration. Moreover, human myocardial concentrations of these compounds and their metabolites remain insufficiently characterized. Formulation-dependent differences further complicate comparisons across studies [72], while the potential for interactions with drug-metabolizing enzymes and transporters warrants dedicated evaluation during concomitant anticancer therapy. Finally, direct mg/kg conversion from animal studies to humans may be misleading; translational assessment should consider species-specific pharmacokinetics, route of administration, formulation and systemic exposure [76].

In addition, products such as blueberry extract and total flavonoids from *Dracocephalum moldavica* are complex multicomponent preparations rather than purified phytochemicals. Therefore, their composition and degree of standardization may vary. This variability limits cross-study comparability and makes it difficult to attribute pharmacological effects to a single active constituent [48,69].

Another limitation across circulating miRNA studies is assay heterogeneity. There are differences in sample collection and processing, RNA extraction methods, quantification platforms, normalization methods and the timing of blood sampling in relation to chemotherapy exposure [18,19,20,21]. Therefore, methodological harmonization is essential to ensure that circulating miRNA candidates can be developed into analytically valid, reproducible and clinically useful biomarkers.

The preclinical evidence should also be interpreted in light of methodological and reporting limitations, as summarized in Supplementary Table S2. Across the studies considered, information on randomization, allocation concealment, blinded outcome assessment and a priori sample-size calculation was not consistently reported. Many in vivo studies used animals of a single sex, while the extent of biological and technical replication varied between experimental models. Moreover, prespecification of outcomes and the potential for selective reporting were generally difficult to assess from the published reports. For miRNA studies, additional sources of variability include the choice of normalization controls and the use, or absence, of correction for multiple comparisons when several miRNAs or molecular endpoints were evaluated. Independent replication of individual mechanistic findings remains limited, and publication bias toward positive cardioprotective results cannot be excluded. These limitations reduce confidence in the robustness and direct translational relevance of individual preclinical findings and reinforce the need for independent replication in rigorously designed models.

Finally, a major translational limitation is that antitumor efficacy is rarely evaluated alongside cardioprotection. Most of the phytochemical studies considered here used non-tumor-bearing models and did not directly assess whether the intervention altered doxorubicin antitumor activity. An exception is the study by Yao et al., in which EGCG was evaluated in Sarcoma-180 tumor-bearing mice. In this model, co-administration of EGCG with doxorubicin did not reduce antitumor activity and was associated with greater inhibition of tumor growth and increased tumor-cell apoptosis compared with doxorubicin alone [63]. Resveratrol has also been examined in cancer-cell proliferation assays in one of the studies discussed here, although preservation of doxorubicin antitumor efficacy was not directly tested in a tumor-bearing model [53]. Overall, evidence regarding effects on doxorubicin pharmacokinetics, intracellular accumulation, drug transporters or metabolic interactions remains very limited in the cardioprotection studies reviewed. Therefore, preservation of antitumor efficacy should be demonstrated directly rather than inferred from cardioprotective or antioxidant effects alone.

## 7. Future Directions

In light of the above, future studies should adopt more rigorous designs. For studies aiming to establish miRNA-mediated cardioprotection, this requires the use of miRNA mimics and/or inhibitors, as well as rescue experiments to demonstrate whether the candidate miRNA is clearly implicated with the observed phenotype. Moreover, future studies should include pharmacokinetic and exposure assessments. These should characterize circulating parent compounds as well as metabolites and, where feasible, myocardial exposure, using clinically relevant formulations and dosing strategies [71,72,73,74,75,76]. These assessments should be integrated with molecular biomarkers and cardiac imaging endpoints such as GLS and LVEF [1,2].

Another evaluation that should be incorporated in subsequent investigations is preservation of antitumor efficacy. Future cardioprotective studies should include tumor-relevant models and directly assess whether the intervention alters doxorubicin efficacy, systemic exposure or tumor response.

**Figure 2:**
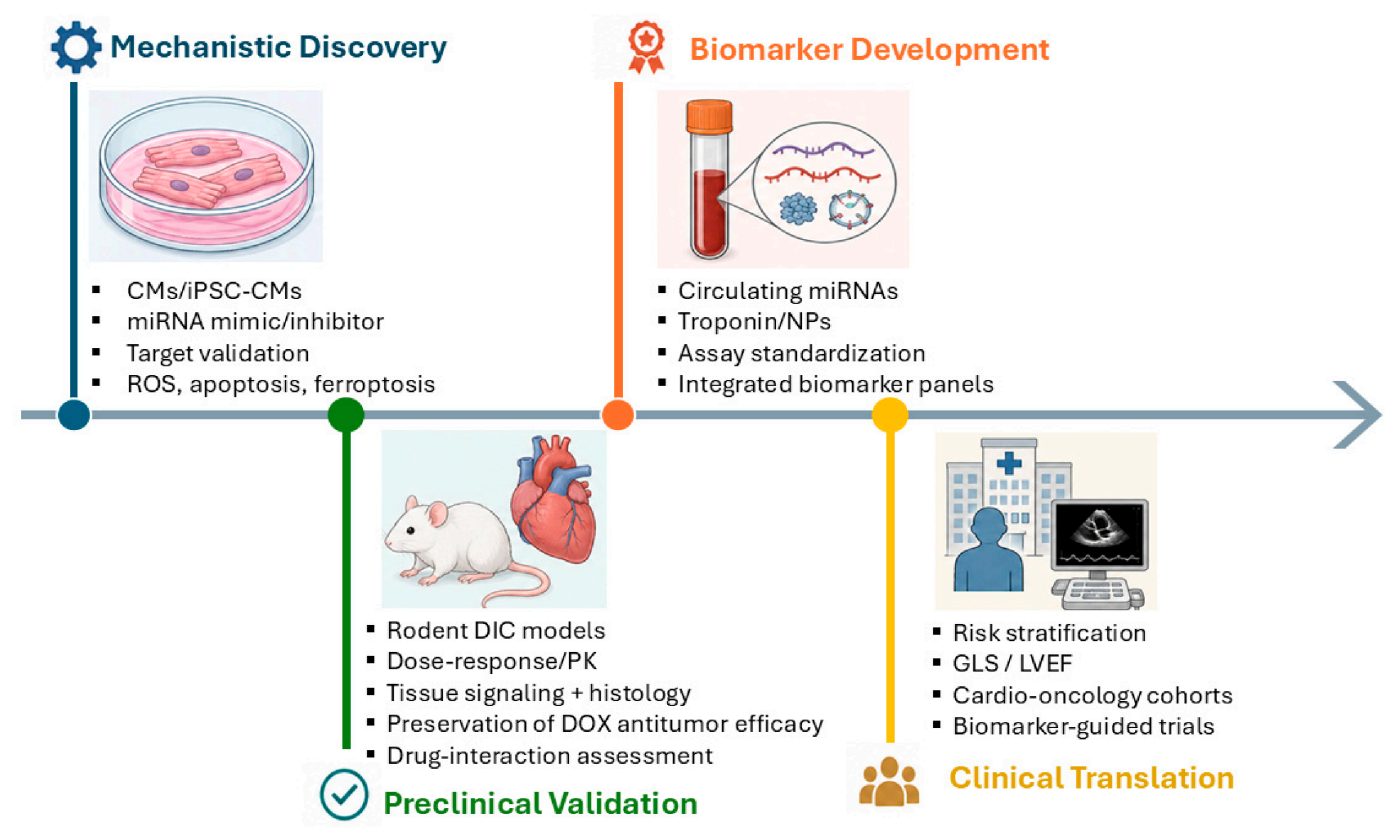
Translational pipeline from in vitro work to cardio-oncology biomarker-guided trials. CMs, cardiomyocytes; DIC, doxorubicin-induced cardiotoxicity; DOX, doxorubicin; GLS, global longitudinal strain; iPSC-CMs, induced pluripotent stem cell-derived cardiomyocytes; LVEF, left ventricular ejection fraction; miRNA, microRNA; NPs, natriuretic peptides; PK, pharmacokinetics; ROS, reactive oxygen species.

To conclude, a phased research strategy should start with the discovery of DIC-relevant miRNA signatures in cellular and small-animal systems. Subsequent steps should include confirmation of miRNA causality, validation of circulating biomarkers in well-defined patient cohorts and, finally, biomarker-guided interventional studies (Figure 2).

## 8. Conclusions

Several miRNAs have experimentally supported mechanistic roles in DIC, whereas their value as circulating biomarkers remains under investigation. Among the selected phytochemical examples discussed in this review, taxifolin provides direct miRNA-linked evidence involving the miR-200a/NRF2 pathway, with functional miRNA involvement demonstrated in vitro. Blueberry extract provides miRNA-associated evidence involving miR-140-5p, *Sirt2* and *Nrf2* expression, although causal miRNA involvement has not been established. The other phytochemical examples discussed here illustrate pathway convergence between experimentally supported cardioprotective mechanisms and miRNA-regulated pathways in DIC, without direct demonstration of miRNA mediation. Therefore, these relationships should be considered as hypothesis-generating rather than validated miRNA-mediated mechanisms. Future studies should adopt more rigorous designs and incorporate pharmacokinetic assessments together with cardio-oncology-specific safety and efficacy endpoints. This approach would help towards the translation of promising preclinical findings into clinically relevant strategies for cardio-oncology practice.

## Figures and Tables

**Table 1:** Selected examples of polyphenol- and flavonoid-mediated cardioprotection in DIC according to their relationship with miRNA-regulated mechanisms.

Compound/Extract	miRNA Directly Assessed in the Phytochemical Study	Functional miRNA Validation	Experimentally Supported Cardioprotective Mechanism	Critical Interpretation	Supporting References
Taxifolin	miR-200a	Yes. miR-200a inhibition was performed in H9c2 cells.	miR-200a/KEAP1/NRF2 signaling; attenuation of oxidative stress and ferroptosis.	Direct miRNA-linked evidence in vitro. Functional experiments support involvement of miR-200a in taxifolin-mediated protection; cardioprotective effects were also demonstrated in vivo.	[42]
Blueberry extract	miR-140-5p	No.	Attenuation of biochemical and histopathological cardiac injury; restoration of *Sirt2* and *Nrf2* expression; miR-140-5p expression was assessed.	miRNA-associated evidence without causal validation. miR-140-5p was measured, but its causal contribution to blueberry-mediated cardioprotection was not functionally tested.	[48]
Quercetin	None	No.	Bmi-1-associated protection; reduction in oxidative stress, mitochondrial dysfunction and apoptosis; improvement in cardiac biochemical, functional and histological parameters.	Pathway-convergent evidence. Cardioprotective mechanisms are experimentally supported, but no specific miRNA was assessed as a mediator of quercetin activity.	[49,50,51,52]
Resveratrol	None	No.	SIRT1-dependent mitochondrial protection; p53 deacetylation; attenuation of apoptosis; modulation of autophagy and SIRT1/NRF2-related signaling.	Pathway-convergent evidence. The experimentally supported pathways overlap with miRNA-regulated mechanisms in DIC, but direct miRNA mediation was not investigated.	[53,54,55,56]
Curcumin	None	No.	Suppression of oxidative stress; preservation of mitochondrial function; 14-3-3γ-associated protection; anti-apoptotic effects.	Pathway-convergent evidence. No compound-specific miRNA-mediated mechanism was demonstrated in the studies considered.	[57,58,59]
EGCG	None	No.	AMPKα2 activation; adaptive autophagy; attenuation of ferroptosis, oxidative stress and mitochondrial injury.	Pathway-convergent evidence. AMPK-related mechanisms are experimentally supported, but miRNA involvement was not directly assessed.	[60,61,62,63]
Isoquercitrin	None	No.	Attenuation of oxidative stress and inflammatory injury; molecular pathways explored using integrated network pharmacology and RNA-seq.	Pathway-convergent evidence. Molecular and transcriptomic effects were identified, but no miRNA-mediated mechanism was directly tested.	[65]
Genistein	None	No.	NRF2/HO-1-associated antioxidant protection; ERK/STAT3/c-Myc-related modulation of autophagy and apoptosis.	Pathway-convergent evidence. Experimentally supported cardioprotective pathways were identified without direct assessment of miRNA involvement.	[66,67]
Engeletin	None	No.	AMPK-dependent attenuation of cardiac dysfunction, oxidative stress, inflammation, apoptosis, autophagy dysregulation and mitochondrial injury.	Pathway-convergent evidence. AMPK involvement was experimentally investigated, but no miRNA was assessed.	[68]
*Dracocephalum moldavica* L. total flavonoids	None	No.	Preservation of mitochondrial homeostasis through AMPK/PGC-1α signaling.	Pathway-convergent evidence. The pathway was experimentally investigated, but direct miRNA involvement was not assessed.	[69]

Abbreviations: 14-3-3γ, 14-3-3 gamma protein; AMPK, AMP-activated protein kinase; AMPKα2, AMP-activated protein kinase alpha 2; Bmi-1, B-cell-specific Moloney murine leukemia virus integration site 1; c-Myc, MYC proto-oncogene protein; DIC, doxorubicin-induced cardiotoxicity; EGCG, epigallocatechin-3-gallate; ERK, extracellular signal-regulated kinase; H9c2, rat cardiomyoblast cell line; HO-1, heme oxygenase 1; KEAP1, Kelch-like ECH-associated protein 1; miRNA, microRNA; *Nrf2*, nuclear factor erythroid 2-related factor 2 gene; NRF2, nuclear factor erythroid 2-related factor 2 protein; p53, tumor protein p53; PGC-1α, peroxisome proliferator-activated receptor gamma coactivator 1-alpha; RNA-seq, RNA sequencing; SIRT1, sirtuin 1 protein; *Sirt2*, sirtuin 2 gene; STAT3, signal transducer and activator of transcription 3. Table footnote. The phytochemicals shown are selected examples discussed in this critical narrative review and do not represent an exhaustive inventory of compounds investigated in DIC. “Direct miRNA-linked” indicates functional investigation of miRNA involvement in the phytochemical study; “miRNA-associated” indicates measurement of a miRNA without functional validation of causality; and “pathway-convergent” indicates overlap between experimentally supported cardioprotective mechanisms and miRNA-regulated DIC pathways without direct assessment of miRNA involvement.

## Data Availability

No new data were created or analyzed in this study.

## Supplementary Materials

The following supporting information can be downloaded at https://www.mdpi.com/article/10.3390/ijms27209224/s1.
