# Supplementary material for: Polyphenols and Flavonoids in microRNA-Regulated Pathways of Doxorubicin-Induced Cardiotoxicity: Mechanistic Evidence, Biomarker Potential and Translational Gaps
Source: Int J Mol Sci. 2026 Oct 17;27(20):9224. doi: 10.3390/ijms27209224 (PMC13704963; doi:10.3390/ijms27209224)
Supplement: Supplementary file 1 [file ijms-27-09224-s001.zip › ijms-4604896-supplementary.pdf]

Supplementary Material

Supplementary Table S1. Methodological and clinical characteristics of primary clinical studies of circulating microRNAs in doxorubicin-treated patients discussed in Section 4.2.

Reference / population & DOX exposure	Circulating miRNA(s)	Matrix / sampling / normalization	Cardiac endpoint(s) & dose relationship	Main findings / diagnostic performance	Independent validation
**[24]**<br>88 women with breast cancer; DOX subgroup n=32 (DOX cTn− n=14; DOX cTn+ n=18). Four anthracycline cycles; cumulative dose reported in DOX equivalents.	Baseline DOX-associated signature: miR-122-5p, miR-499a-5p and miR-885-5p. Treatment-related changes also examined for miR-34a-5p, miR-122-5p and miR-885-5p.	Plasma. Blood collected at baseline (T0), during treatment (T1; up to 1 month after last infusion) and follow-up (T2; 3–12 months after last infusion). OpenArray screening: Global Mean Normalization; single-assay validation: miR-16 reference.	Primary endpoint: cTn positivity; echocardiographic LVEF also assessed. In the DOX subgroup, cumulative anthracycline exposure did not differ significantly between cTn− and cTn+ groups (240±0 vs 226.2±38.7 mg/m² DOX equivalents; p=0.43). No relevant LVEF decline occurred during 12-month follow-up.	At baseline, miR-122-5p, miR-499a-5p and miR-885-5p were higher in DOX cTn+ than DOX cTn− patients. The three-miRNA logistic model yielded AUC 0.79 for subsequent cTn elevation. Sensitivity/specificity were not reported as a validated clinical cut-off.	Internal assay validation within the same cohort; no independent external cohort.
**[42]**<br>56 women with breast cancer from the placebo arm of CECCY; DOX 60 mg/m² + cyclophosphamide 600 mg/m² every 3 weeks ×4 (planned cumulative DOX 240 mg/m²), followed by taxane.	miR-1, miR-133b, miR-146a, miR-423-5p; miR-208a/b were undetectable.	Plasma. Blood collected 3 weeks after each DOX cycle. RT-qPCR; exogenous cel-miR-39 normalization.	cTnI and serial LVEF. Cardiotoxicity: LVEF reduction ≥10% and/or LVEF <50%. Fixed four-cycle DOX regimen; no formal continuous miRNA–dose analysis.	miR-1 was associated with change in LVEF (r=−0.531, p<0.001). For cardiotoxicity discrimination, miR-1 AUC was 0.851 (95% CI 0.729–0.933); sensitivity/specificity were not reported.	No independent external cohort.
**[43]**<br>12 women with invasive ductal breast carcinoma: 6 DOX-cardiotoxicity cases and 6 DOX-treated controls. Mean DOX dose 341.3±94.2 vs 379.2±12.8 mg/m², respectively (p=0.353).	miR-133a-3p, miR-133b, miR-15a-5p, miR-208a-5p and miR-499a-5p.	Serum. Sampling before chemotherapy (T0) and at the end of chemotherapy, up to 7 days after completion (T1). RT-qPCR; RNU43 internal control.	LVEF by echocardiography, cTnI and NT-proBNP. Cardiotoxicity defined by LVEF criteria and/or ≥20% increase in cTnI and NT-proBNP from baseline. No dose–miRNA correlation reported.	miR-133a was 12.3-fold higher at T1 in cardiotoxicity cases than controls; no baseline difference. No ROC/AUC, sensitivity, specificity or diagnostic threshold was reported.	No independent external cohort.
**[44]**<br>45 women with non-metastatic breast cancer with complete paired data; dose-dense DOX 60 mg/m² + cyclophosphamide 600 mg/m² every 2 weeks ×4.	miR-199a.	Plasma. Sampling at baseline and after completion of four DOX/cyclophosphamide cycles. qRT-PCR; U6 snRNA endogenous reference; no exogenous spike-in control.	cTnI, NT-proBNP and echocardiographic LVEF. Cumulative DOX dose was inversely correlated with ΔLVEF (ρ=−0.424, p=0.004) but not with ΔmiR-199a.	miR-199a and cTnI increased significantly after treatment (both p<0.001), whereas NT-proBNP and cohort-level LVEF did not. ΔmiR-199a was not significantly associated with contemporaneous ΔLVEF. No ROC/AUC, sensitivity or specificity was reported.	No independent external cohort.

Abbreviations: AUC, area under the receiver operating characteristic curve; CECCY, Carvedilol Effect in Preventing Chemotherapy-Induced Cardiotoxicity; cel-miR-39, *Caenorhabditis elegans* miR-39; CI, confidence interval; cTn, cardiac troponin; cTnI, cardiac troponin I; DOX, doxorubicin; LVEF, left ventricular ejection fraction; miRNA, microRNA; NT-proBNP, N-terminal pro-B-type natriuretic peptide; qRT-PCR, quantitative reverse-transcription polymerase chain reaction; RNU43, small nucleolar RNA U43; ROC, receiver operating characteristic; RT-qPCR, reverse-transcription quantitative polymerase chain reaction; snRNA, small nuclear RNA; U6, U6 small nuclear RNA; Δ, change from baseline.

Table note: Reference [24] included both doxorubicin- and epirubicin-treated patients; only findings from the doxorubicin-treated subgroup relevant to the present review are summarized here.

Table S2. Experimental characteristics of the phytochemical studies discussed in this review.

Study / ref.	Compound / form	Experimental model	Species / strain / sex or cell type	DOX regimen	Phytochemical intervention	Design	miRNA assessment / analytical details / functional manipulation	Main cardiac endpoints	Antitumor efficacy assessed	Key limitations
[45]	Taxifolin (TAX); purified compound; purity NR	Acute in vivo DIC + H9c2 model	Male C57BL/6 mice, 6-8 wk; H9c2 cells	Mouse: 10 mg/kg i.p., single dose after 7 d pretreatment; H9c2: DOX exposure in cell model (1 μM used in mechanistic experiments)	Mouse: TAX 20 mg/kg/day by gavage for 14 days, starting 7 days before DOX administration; H9c2: TAX 10 μM pretreatment for 12 h, followed by co-treatment with DOX 1 μM for 24 h..	Preventive pretreatment with continued taxifolin administration after DOX exposure	miR-200a quantified by qRT-PCR (Bulge-Loop miRNA kit), with U6 as the internal control; miR-200a inhibitor and *Nrf2* siRNA used for functional testing in H9c2 cells	LVEF/LVFS, serum LDH/CK-MB/cTnI, histology, ROS/oxidative stress, iron/ferroptosis markers, NRF2/HO-1/NQO1	No	Single-dose DOX model; functional miRNA validation confined to the cellular component; non-tumor-bearing animals
[49]	Blueberry extract; commercial extract; composition/standardization incompletely reported	Acute in vivo DIC	Wistar rats, 8/group; sex NR	18 mg/kg i.p., single dose on day 11	Blueberry extract 80 mg/kg orally daily for 14 d	Preventive pretreatment with continued exposure	Rat cardiac-tissue miR-140-5p quantified by qRT-PCR after miRNeasy RNA extraction and SYBR Green amplification; normalization strategy not specified in the accessible article; no gain-/loss-of-function or rescue experiment	cTnT, NT-proBNP, MPO, MDA, GSH, SOD, TOP II, histopathology, miR-140-5p/***Sirt2*/*Nrf2*** expression	No	Single high-dose DOX model; complex extract; no functional miRNA validation; extract standardization limited
[50]	Quercetin; purified compound; purity NR	Acute in vivo DIC + H9c2 model	Female C57BL/6 mice, 8-10 wk; H9c2 cells	Mouse: 20 mg/kg i.p. single dose for biochemical/survival studies; 10 mg/kg i.p. for echocardiography; H9c2: 0-16 μM for 48 h	Mouse: quercetin 100 mg/kg p.o. once daily for 10 d, with 5 d pretreatment before DOX; H9c2: 50 or 100 μM for 48 h	Preventive pretreatment with continued treatment	No miRNA assessment; *Bmi-1* siRNA/overexpression used for pathway validation	Survival, LVEF/LVFS, CK/LDH, apoptosis, ROS/MDA/SOD, mitochondrial function, DNA damage, Bmi-1 signaling	No	Acute/high-dose DOX model; high quercetin exposure; no tumor-bearing model; pathway validation was non-miRNA
[51]	Quercetin; purified compound; purity NR	Acute in vitro primary cardiomyocyte model	Primary neonatal Sprague-Dawley rat cardiomyocytes	1 μM for 24 h	Quercetin 10, 20, 40 or 80 μM; 22 h pretreatment before DOX	Preventive pretreatment	No miRNA assessment; 14-3-3γ knockdown used for pathway validation	Cell viability, LDH, caspase-3/apoptosis, ROS/MDA, SOD/CAT/GPX, GSH/GSSG, MMP, mPTP	No	In vitro only; neonatal cardiomyocytes; no tumor context; no miRNA testing
[52]	Quercetin; purified compound; purity NR	Repeated-dose in vivo DIC	Wistar rats, 150-250 g, 8/group; sex NR	2.5 mg/kg i.p. every 48 h × 6; cumulative 15 mg/kg over 2 wk	Quercetin 50 or 100 mg/kg/day by oral gavage during the 2-wk DOX protocol	Concurrent cardioprotection in repeated-dose model	No miRNA assessment or manipulation	Echocardiography, NT-proBNP, TnI, CK-MB, SOD1/ NRF2, γH2AX/8-OHdG, oxidative damage, cardiac ultrastructure	No	Non-tumor-bearing model; sex not reported in methods; no miRNA or antitumor assessment

Study / ref.	Compound / form	Experimental model	Species / strain / sex or cell type	DOX regimen	Phytochemical intervention	Design	miRNA assessment / analytical details / functional manipulation	Main cardiac endpoints	Antitumor efficacy assessed	Key limitations
[53]	Quercetin; purified compound; purity >95% (HPLC grade).	Repeated-dose in vivo DIC	Adult male Wistar albino rats, 3-4 months, 220-250 g; n=6/group (24 total).	2.5 mg/kg i.p. every 2 days ×7 over 2 weeks; cumulative 17.5 mg/kg.	Quercetin 80 mg/kg/day orally by gavage for 2 weeks.	Concurrent administration; randomized, blinded and allocation-concealed.	No miRNA assessment.	LDH, CPK, MDA, NO, GPX; histology/fibrosis; 8-OHdG and p53 immunohistochemistry; TEM/ultrastructure.	No	Small sample size; absence of functional cardiac assessment; no Western blot/qPCR confirmation of p53-related signaling; limited specificity of morphology-based fibroblast identification; no tumor-bearing or anticancer-response experiments; no miRNA assessment.
[54]	Resveratrol (RES); purified compound; studied alone and with FGF1	Acute in vivo DIC + H9c2 model; cancer-cell proliferation assays	Male C57BL/6J mice, 8 wk, 6/group; H9c2 cells; HepG2, 5637 and MCF-7 cancer cells	Mouse: 20 mg/kg i.p., single dose after 7 d pretreatment; H9c2: 1 μM for 24 h	Mouse: RES 10 mg/kg/day i.p. for 7 d ± FGF1 0.5 mg/kg/day; H9c2: RES 20 μM ± FGF1 100 ng/mL for 24 h	Preventive pretreatment; mechanistic evaluation included RES alone and RES+FGF1	No miRNA assessment; *Sirt1* and *Nrf2* shRNAs used to test pathway dependence	Oxidative stress, apoptosis, histology/biochemistry, SIRT1/NRF2 signaling	No direct DOX antitumor-efficacy test; RES reduced FGF1 growth-promoting activity in cancer cells	Acute single-dose DOX model; major mechanistic conclusions involve RES+FGF1 co-treatment; no tumor-bearing animal model
[55]	Resveratrol; purified compound	Acute in vitro cardiomyocyte model	Neonatal rat ventricular cardiomyocytes	DOX 50 μM for 10 min in mitochondrial ROS assays and 1 μM for 24 h in cell-death assays	Resveratrol 10 μM for 72 h before DOX	Prolonged preventive pretreatment	No miRNA assessment; SIRT1 dependence tested pharmacologically with nicotinamide	Mitochondrial ROS, MnSOD, mitochondrial membrane potential/function, cardiomyocyte death, SIRT1 activity	No	In vitro only; neonatal cells; prolonged pretreatment; no tumor context
[56]	Resveratrol; purified compound	Acute in vitro H9c2 + short-duration in vivo rat DIC	H9c2 cells; Sprague-Dawley rats, 8-10 wk; sex NR	H9c2: 2 μM for 24 h; Rats: DOX 15 mg/kg i.p.; dosing frequency was inconsistently reported in the original article (Methods: 15 mg/kg/day for 1 week; Results/Figure 4: 15 mg/kg).	H9c2: RES 20 μM for 24 h; rats: RES 10 mg/kg/day i.p. for 1 wk	Concurrent co-treatment with mechanistic AMPK inhibition	No miRNA assessment; Compound C used to probe AMPK-dependent autophagy	Apoptosis, autophagy, p-AMPK/mTOR/ULK1, p38MAPK/p53, LC3, caspase-3, TUNEL	No	Short-duration model; very high in vivo DOX exposure as reported; sex NR; no tumor-bearing assessment
[57]	Resveratrol; purified compound	Longer-term repeated-dose in vivo DIC	Male BALB/c mice, 8 wk, 10/group	8 mg/kg i.p. every 3 wk; cumulative 24 mg/kg	Resveratrol incorporated into chow at 0.01% w/w (~15 mg/kg/day) for 7 wk	Concurrent long-term dietary resveratrol exposure	No miRNA assessment; SIRT1-p53 interaction, p53 deacetylation and Bax signaling examined	LV hemodynamics, LDH, protein carbonyls, MDA, TUNEL, SIRT1/p53/Bax, cytochrome c release	No	Non-tumor-bearing model; dietary exposure without PK assessment; no miRNA testing

Study / ref.	Compound / form	Experimental model	Species / strain / sex or cell type	DOX regimen	Phytochemical intervention	Design	miRNA assessment / analytical details / functional manipulation	Main cardiac endpoints	Antitumor efficacy assessed	Key limitations
[59]	Curcumin dry extract >96% (lot reported)	Acute in vivo DIC	Adult male BALB/c mice, 30-35 g	Single 9 or 16 mg/kg i.p. dose on day 0	Curcumin 100 mg/kg/day by gavage for 15 d, beginning 2 h after DOX	Post-exposure treatment	No miRNA assessment or manipulation	Survival, histopathology, ROS, lipid peroxidation, sulfhydryls, protein carbonyls, antioxidant markers	No	Single-dose DOX model; non-tumor-bearing animals; mechanistic focus mainly oxidative stress
[60]	Curcumin; purity ≥98%	Repeated-dose in vivo mouse DIC + acute primary cardiomyocyte model	Adult Kun-Ming mice; primary cardiomyocytes from 2-day-old Sprague-Dawley rats	In vivo: 2.5 mg/kg i.p. × 6 over 3 wk; cumulative 15 mg/kg; in vitro: 1 µM for 24 h	In vivo: 50 mg/kg/day intragastrically for 6 wk, given 1 h before DOX; in vitro: 5–40 µM for 22 h before DOX	Prolonged preventive pretreatment	No miRNA assessment; 14-3-3γ knockdown used in vivo and in vitro	ECG, LDH/CK, histology, apoptosis/caspase-3, ROS/MDA, antioxidant enzymes, MMP, mPTP, 14-3-3γ/Bad/Bcl-2	No	Long pretreatment and high experimental exposures; no tumor-bearing model; no miRNA testing
[61]	EGCG; purified green-tea catechin; purity NR	Acute in vitro cardiomyocyte model	Primary neonatal Sprague-Dawley rat cardiomyocytes	0.1, 0.5 or 1 µM; 1 µM used in subsequent experiments, typically 24 h	EGCG 3.13–200 µM dose-ranging; 38, 50 and 100 µM used in mechanistic experiments; 1 h pretreatment	Preventive pretreatment	No miRNA assessment	Cell viability/death, LDH, ROS, MDA, MnSOD/CAT/ GPX expression and activity, apoptosis	No	In vitro only; neonatal cardiomyocytes; high micromolar EGCG concentrations; no tumor context
[62]	EGCG; purified compound	Repeated-dose in vivo DIC + acute NRCM/H9c2 models	Male C57BL/6 mice, 8 wk; neonatal rat cardiomyocytes; H9c2 cells	In vitro: 1 µM for 48 h; in vivo: 2.5 mg/kg i.p. × 6 over 3 wk; cumulative 15 mg/kg	In vitro: EGCG 20 µM for 24 h before DOX; in vivo: EGCG 20 mg/kg/day by oral gavage for 6 wk; during the 3-wk DOX treatment period, DOX was administered 1 h before EGCG.	Preventive pretreatment with continued administration during DOX exposure	No miRNA assessment; pAD/AMPK-shRNA, Compound C and autophagy inhibitors used for pathway validation	Ferroptosis, iron, ROS/lipid peroxidation, autophagy, AMPKα2/mTOR/ULK1, mitochondrial function, LVEF/LVFS, histology	No	Multiple models but no tumor-bearing model; miRNA mediation not examined; long pretreatment
[63]	EGCG; purified compound; purity NR	Acute screening + repeated-dose mechanistic in vivo DIC	Male Wistar rats, 200–250 g	Dose-screening: single 15 mg/kg i.p. dose; mechanistic protocol: 2.5 mg/kg i.p. on alternate days during weeks 3-4 to cumulative 15 mg/kg	Dose-screening: EGCG 10, 20 or 40 mg/kg/day by oral gavage for 12 d; mechanistic protocol: 40 mg/kg by oral gavage 3×/wk for 4 wk	Preventive pretreatment	No miRNA assessment	ECG, CK-MB/LDH, histology, GSH/lipid peroxidation/antioxidant enzymes, ErbB2, NF-κB, p53, calpain-2, caspase-3/12, Hsp70	No	Non-tumor-bearing model; no direct antitumor-efficacy or miRNA assessment
[64]	EGCG; Sigma reagent; purity NR	Short-duration repeated-dose tumor-bearing in vivo DIC	Sarcoma-180 tumor-bearing Kunming mice, 22 ± 2 g; sex not reported	2 mg/kg/day i.p. for 10 consecutive d	EGCG 25 mg/kg/day i.p. for 10 consecutive d, alone or with DOX	Concurrent treatment in tumor-bearing animals	No miRNA assessment	Tumor weight/apoptosis; serum LDH; cardiac ultrastructure; cardiomyocyte apoptosis, ROS, MMP, Ca2+, MnSOD	Yes. EGCG+DOX inhibited tumor growth and increased tumor-cell apoptosis versus DOX alone	Single S180 tumor model; no miRNA testing; short treatment period

Study / ref.	Compound / form	Experimental model	Species / strain / sex or cell type	DOX regimen	Phytochemical intervention	Design	miRNA assessment / analytical details / functional manipulation	Main cardiac endpoints	Antitumor efficacy assessed	Key limitations
[66]	Isoquercitrin (IQC); purified compound; purity NR	Acute in vitro human cardiomyocyte model + network pharmacology/RNA-seq	Human AC16 cardiomyocytes	0.2 μM for 48 h	IQC 0-300 μM with DOX for 48 h; 100 and 300 μM used for key validation experiments	Concurrent treatment	No miRNA assessment; RNA-seq, RT-qPCR and computational target analyses	Cell viability/death, inflammatory/oxidative-stress pathways, RNA-seq hub genes including *CCL19* and *PADI4*	No	In vitro only; high micromolar IQC exposure; substantial computational component; no tumor or miRNA assessment
[67]	Genistein; purified compound; purity NR	Repeated-dose in vivo DIC	Male 129/Sv mice, 12–14 wk	5 mg/kg i.p. once weekly after 1 wk of genistein pretreatment, continued through the 4-wk study period	Genistein 5 mg/kg i.p. for 4 wk	Preventive pretreatment followed by concurrent weekly DOX exposure	No miRNA assessment; NRF2/HO-1/NQO1 and apoptotic signaling examined	Cardiac troponin, ROS/LPO/4-HNE, inflammatory cytokines, NRF2/HO-1/NQO1, Akt/Bcl-2/Bax/caspase-3	No	Non-tumor-bearing mouse model; single genistein dose; no miRNA or antitumor-efficacy assessment
[68]	Genistein; purified compound; assay >99% in animal experiments and >99.9% for cell-culture reagents	Repeated-dose in vivo rat DIC + acute H9c2 model	Male Sprague-Dawley rats, 180–200 g; H9c2 cells	In vivo: 3 mg/kg i.p. every 7 d × 6; cumulative 18 mg/kg; in vitro: 5 μM for 24 h	In vivo: genistein 20 or 40 mg/kg/day subcutaneously for 6 wk; in vitro: dose-ranging genistein pretreatment for 30 min before DOX	Preventive pretreatment with continued genistein exposure during the DOX course	No miRNA assessment; ERK/STAT3/c-Myc signaling examined; U0126 (MEK1/2 inhibitor) used for mechanistic validation	Hemodynamics, histopathology/TEM, apoptosis, autophagy, ERK/STAT3/c-Myc signaling, H9c2 viability	No	Non-tumor-bearing model; male-only animals; higher genistein dose (40 mg/kg/day) was less protective and showed potentially adverse cardiac effects; no miRNA assessment
[69]	Engeletin (ENG); MedChemExpress reagent; purity NR	Chronic in vivo DIC + acute H9c2 model	Male C57BL/6 mice, 8 wk, n=5/group; H9c2 cells	In vivo: 5 mg/kg i.p. once weekly for 5 wk; in vitro: 1 μM for 24 h	In vivo: ENG 20 mg/kg/day i.p. for 14 days before initiation of DOX treatment; in vitro: 80 μM, 2 h pretreatment then co-treatment with DOX for 24 h	Preventive pretreatment	No miRNA assessment; Compound C used to test AMPK dependence	LVEF/LVFS/LVEDd/LVESd, BNP, MDA/4-HNE, histology/fibrosis, apoptosis, inflammation, autophagy, mitochondrial injury, AMPK	No	Small in vivo groups (n=5); non-tumor-bearing; male-only; no miRNA testing
[70]	Total flavonoids from *Dracocephalum moldavica* L. (TFDM); complex standardized flavonoid preparation	Short-duration repeated-dose in vivo DIC + acute H9c2 model	Male C57BL/6 mice, 8 wk; H9c2 cells	In vivo: 20 mg/kg i.p. twice, 3 d apart after 7 d pretreatment; in vitro: 1 μM for 24 h	In vivo: TFDM 45, 90 or 180 mg/kg/day by gavage for 14 d; in vitro: 1.5625-200 μg/mL dose-ranging, 6 h pretreatment then DOX 24 h	Preventive pretreatment with continued TFDM administration during DOX exposure	No miRNA assessment; *AMPKα* siRNA and Compound C used for pathway validation	Echocardiography, LDH/CK-MB, oxidative stress, TEM, apoptosis, mitochondrial respiration/homeostasis, AMPK/PGC-1α/NRF1/TFAM	No	Complex mixture rather than single compound; individual-flavonoid exposure uncertain; non-tumor-bearing; no miRNA assessment

Abbreviations: 4-HNE, 4-hydroxynonenal; 8-OHdG, 8-hydroxy-2′-deoxyguanosine; 14-3-3γ, 14-3-3 gamma protein; 5637, human bladder carcinoma cell line; AC16, human ventricular cardiomyocyte cell line; Akt, protein kinase B; AMPK, AMP-activated protein kinase; *AMPKα*, AMP-activated protein kinase alpha subunit gene; AMPKα2, AMP-activated protein kinase alpha 2; Bad, Bcl-2-associated agonist of cell death; Bax, Bcl-2-associated X protein; Bcl-2, B-cell lymphoma 2; Bmi-1, B-cell-specific Moloney murine leukemia virus integration site 1; BNP, B-type natriuretic peptide; CAT, catalase; *CCL19*, C-C motif chemokine ligand 19 gene; CK, creatine kinase; CK-MB, creatine kinase-MB; CPK, creatine phosphokinase; c-Myc, MYC proto-oncogene protein; cTnI, cardiac troponin I; cTnT, cardiac troponin T; DIC, doxorubicin-induced cardiotoxicity; DOX, doxorubicin; ECG, electrocardiography; EGCG, epigallocatechin-3-gallate; ENG, engeletin; ErbB2, erb-

b2 receptor tyrosine kinase 2; ERK, extracellular signal-regulated kinase; FGF1, fibroblast growth factor 1; GPX, glutathione peroxidase; GSH, reduced glutathione; GSSG, oxidized glutathione; H9c2, rat cardiomyoblast cell line; HepG2, human hepatocellular carcinoma cell line; HO-1, heme oxygenase 1; HPLC, high-performance liquid chromatography; Hsp70, heat shock protein 70; i.p., intraperitoneal; IQC, isoquercitrin; LC3, microtubule-associated protein 1 light chain 3; LDH, lactate dehydrogenase; LPO, lipid peroxidation; LVEDd, left ventricular end-diastolic diameter; LVEF, left ventricular ejection fraction; LVESd, left ventricular end-systolic diameter; LVFS, left ventricular fractional shortening; MCF-7, human breast adenocarcinoma cell line; MDA, malondialdehyde; MEK1/2, mitogen-activated protein kinase kinase 1/2; miRNA, microRNA; MMP, mitochondrial membrane potential; MnSOD, manganese superoxide dismutase; MPO, myeloperoxidase; mPTP, mitochondrial permeability transition pore; mTOR, mechanistic target of rapamycin; NF-κB, nuclear factor kappa B; NO, nitric oxide; NQO1, NAD(P)H quinone dehydrogenase 1; NR, not reported in the accessible primary source; NRCM, neonatal rat cardiomyocyte; NRF1, nuclear respiratory factor 1; *Nrf2*, nuclear factor erythroid 2-related factor 2 gene; NRF2, nuclear factor erythroid 2-related factor 2 protein; NT-proBNP, N-terminal pro-B-type natriuretic peptide; p-AMPK, phosphorylated AMP-activated protein kinase; p38MAPK, p38 mitogen-activated protein kinase; *PADI4*, peptidyl arginine deiminase 4 gene; PGC-1α, peroxisome proliferator-activated receptor gamma coactivator 1-alpha; p.o., oral administration; qPCR, quantitative polymerase chain reaction; qRT-PCR, quantitative reverse-transcription polymerase chain reaction; RES, resveratrol; RNA-seq, RNA sequencing; ROS, reactive oxygen species; RT-qPCR, reverse-transcription quantitative polymerase chain reaction; S180, sarcoma 180; shRNA, short hairpin RNA; siRNA, small interfering RNA; *Sirt1*, sirtuin 1 gene; SIRT1, sirtuin 1 protein; *Sirt2*, sirtuin 2 gene; SOD, superoxide dismutase; SOD1, superoxide dismutase 1; STAT3, signal transducer and activator of transcription 3; TAX, taxifolin; TEM, transmission electron microscopy; TFAM, mitochondrial transcription factor A; TFDM, total flavonoids from *Dracocephalum moldavica* L.; TnI, troponin I; TOP II, topoisomerase II; TUNEL, terminal deoxynucleotidyl transferase dUTP nick-end labeling; U6, U6 small nuclear RNA; ULK1, unc-51-like autophagy activating kinase 1.

Table note: The table summarizes the primary phytochemical studies discussed in the main manuscript. "Antitumor efficacy assessed" refers specifically to experimental assessment of tumor growth, tumor-cell apoptosis, or preservation of doxorubicin antitumor activity within the cited study. The table is descriptive and does not constitute a formal risk-of-bias assessment.
